# Synthesis Antimicrobial and Anticancer Evaluation of 1-Aryl-5-(*o*-methoxyphenyl)-2-*S*-benzyl Isothiobiurets

**DOI:** 10.1155/2014/352626

**Published:** 2014-11-20

**Authors:** Mohammed M. Ansari, Shirish P. Deshmukh, Rizwan Khan, Mohammed Musaddiq

**Affiliations:** ^1^P.G. Department of Chemistry, Shri Shivaji College, Akola, Maharashtra 444001, India; ^2^P.G. Department of Microbiology, Shri Shivaji College, Akola, Maharashtra 444001, India

## Abstract

A series of *S*-benzyl aryl thiourea were condensed with *o*-Methoxy phenyl isocyanate to yield respective isothiobiuret derivatives. The newly synthesized compounds were characterized by ^1^H-NMR, IR, and Mass Spectral studies and tested for biological activities.

## 1. Introduction

Thiourea and its derivatives such as thioureides possess interesting biological properties such as antibacterial [1–3], herbicidal, and fungicidal [4]. Many thiourea show* in-vivio* and* in-vitro* activity against HIV [5]. An impressive number of currently used drugs can be regarded as thiourea derivatives for example, thyreostatic: carbimazole, propylthiouracil, methylthiouracil, and ultrashortnarcotic: thiamylal. Thiourea shows considerable toxicity towards higher organisms and is used as insecticide [6] and rodenticide [7]. Thiourea derivatives find widespread uses in mining industry as floating aid for sulfidic ores [8].

Thiobiurets (mono and di) are important derivatives of (thio) urea which can increase the biological activity of (thio) ureas. Mono and dithiobiuret derivatives are effective bactericide, fungicide, herbicide, and miticides [9]. Dithiobiuret derivatives are used for repelling birds, rodents, leporine animals, and ruminants [10]. 1-Allyl-2-thiobiuret regulates the growth of germinating wheat and cucumber seeds [11]. Oliver and coworkers [12, 13] reported chemosterilising action of dithiobiuret derivatives in male house flies. Mono and dithiobiuret showed effective growth regulating activity [14]. Thiobiuret derivatives also showed analgesic [15], anticonvulsant, and hypnotic activity [16]. Glycosyl urea and their biuret derivatives are reported as potential glycoenzyme inhibitors [17]. Dandale and Deshmukh [18] reported antibacterial and antifungal activities of per O-acetylated lactosyl monothiobiurets.

In quest for biologically more potent compounds, we envisioned synthesizing series of isothiobiuret compounds by reacting* S*-benzyl arylthiourea with* o*-methoxy phenyl isocyanate and studied their antibacterial and anticancer activities.

## 2. Result and Discussion

### 2.1. Chemistry

#### 2.1.1. Experimental

Melting points were recorded on electrothermal melting point apparatus. IR spectra were recorded on a Shamazdu FTIR spectrometer. ^1^H NMR was obtained on a Bruker DRX-300 (300 MHz FT NMR) NMR spectrometer in CDCl_3_ solution with TMS as an internal reference. The mass spectra were recorded on a Jeol SX-102 FAB mass spectrometer. Purity of the compounds was checked by thin layer chromatography using Merck silica gel coated aluminum plates and petroleum ether: ethyl acetate as eluent.

### 2.2. General Scheme of Synthesis

**Step 1 .**

**Scheme 1:**
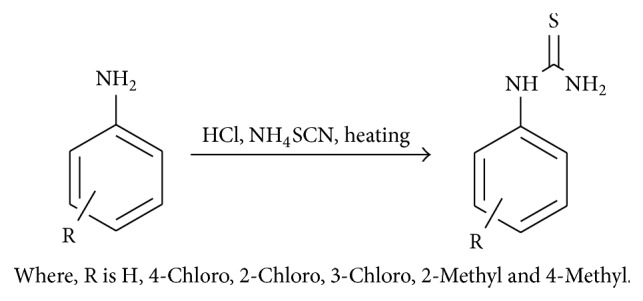


Thiourea synthesis: (see Scheme 1).

**Step 2 .**

**Scheme 2:**
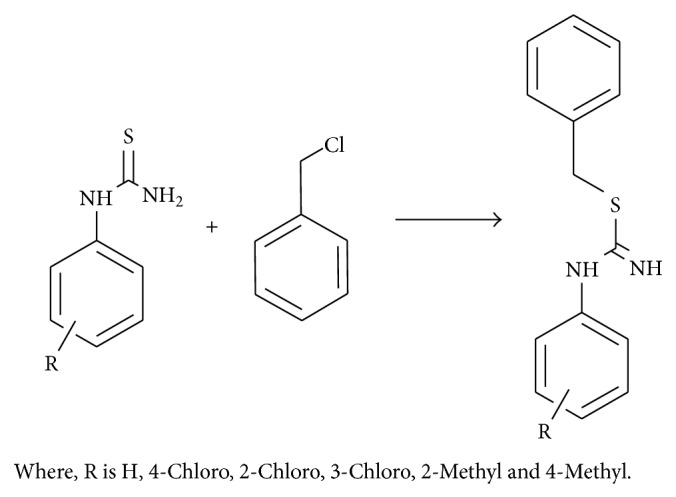


*S*-Benzylation: (see Scheme 2).

**Step 3 .**

**Scheme 3:**
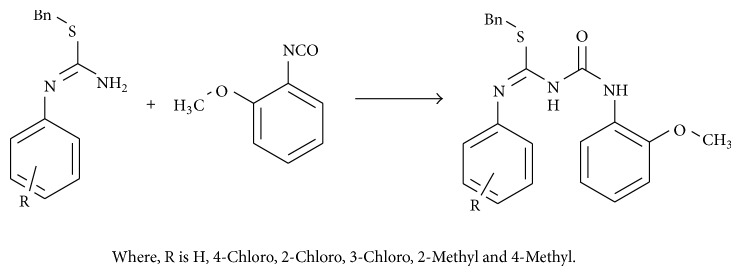


Thiourea isocyanate condensation: (see Scheme 3).

### 2.3. Synthetic Procedure

#### 2.3.1. Synthesis of Aryl* S*-benzyl Thiourea

General synthetic procedure for preparation of Aryl thiourea exemplified by phenyl thiourea: Aniline (30 g, 0.32 mol) was taken in a round bottom flask and, to this, concentrated hydrochloric acid (32.19 mL, 0.32 mol) was added dropwise with continues stirring. After 20 min turbidity appeared and 100 mL of water was added followed by a solution of ammonium thiocyanate (29.42 g, 0.38 mol) in 50 mL of water. This reaction mixture was heated till the solution starts becoming turbid, heating discontinued, reaction mass was poured in ice cold water, and precipitate formed was filtered off and dried. Crude product was recrystallized by hot water, yield (39.22 g, 80%), m.p. 152°C.

General synthetic procedure for preparation of Aryl* S*-benzyl thiourea exemplified by phenyl* S*-benzyl thiourea (II): phenyl thiourea (35 g, 0.23 mol) was dissolved in 75 mL of ethanol and, to this reaction, mass benzyl chloride (29.11 mL, 0.25 mol) was added; once the exotherm subsides, reaction mass was gently heated to reflux and refluxed for 90 min. This reaction mixture was cooled to room temperature and basified under cold condition with ammonia solution, and, on standing, it yields phenyl* S*-benzyl thiourea. Yield (29.2 g, 52.4%), m.p. 75°C.

2-Methoxy phenyl isocyanate was procured from Sigma Aldrich.

General synthetic procedure for preparation of 1-Aryl-5-(*o-*methoxyphenyl)-2-*S*-benzyl isothiobiuret was exemplified using 1-Phenyl-5-(*o-*methoxyphenyl)-2-*S*-benzyl isothiobiuret.

*1-Phenyl-5-(o-methoxyphenyl)-2-S*-*benzyl Isothiobiuret.* Isothiobiuret was synthesized by condensing Phenyl* S*-benzyl thiourea (0.70 g, 2.89 mmole) with* o*-Methoxy phenyl isocyanate (0.43 g, 2.89 mmole) at room temperature in benzene overnight. Solvent was removed by distillation. Solid mass obtained was triturated with pet ether, to afford off-white solid.

*Molecule Number 1: 1-Phenyl-5-(o-methoxyphenyl)-2-S-benzyl Isothiobiuret.* Obtain as off white solid (87.12%) m.p. 100–102°C, TLC *R*_*f*_ 0.7 in EtOAc: Petether (3 : 7) visualized using iodine, IR (KBr) in cm^−1^ *υ* 3290 (N–H); 2906 (Ar–H); 2839 (Methoxy); 1670 (C=O); 1236 (C–N). ^1^H-NMR (300 MHz, CDCl_3_) *δ*: 3.9 (s, 3H), 4.4 (s, 2H), 6.9–7.8 (m, 14H), 8.3 (s, 1H), 11.8 (s, 1H). MS, m/z: 392 [M^+^+1], Anal. Calcd. for C_22_H_21_N_3_O_2_S, Requires: C: 67.50%, H: 5.41%, N: 10.73%, S: 8.19% Found: C: 67.71%, H: 5.21%, N: 10.00%, S: 8.00%.

*Molecule Number 2: 1-(p-Tolyl)-5(o-methoxyphenyl)-2-S-benzyl Isothiobiuret.* Obtain as off white solid (60.06%) m.p. 105–108°C, TLC *R*_*f*_ 0.7 in EtOAc: Petether (3 : 7) visualized using iodine, IR (KBr) in cm^−1^ *υ* 3300 (N–H); 2960 (Ar–H); 2839 (Methoxy); 1741 (C=O). ^1^H-NMR (300 MHz, CDCl_3_) *δ*: 2.4 (s, 3H), 3.8 (s, 3H), 4.5 (s, 2H), 6.89 (d, 2H), 7.0–7.6 (m, 11H), 8.1 (s, 1H), 11.5 (s, 1H). MS, m/z: 406 [M^+^+1], Anal. Calcd. for C_23_H_23_N_3_O_2_S, Requires: C: 68.12%, H: 5.72%, N: 10.36%, O: 7.89%, S: 7.91% Found: C: 67.10%, H: 5.15%, N: 10.00%, S: 8.00%.

*Molecule Number 3: 1-(o-Tolyl)-5-(o-methoxyphenyl)-2-S-benzyl Isothiobiuret.* Obtain as off white solid (60.50%) m.p. 95–97°C, TLC *R*_*f*_ 0.7 in EtOAc: Petether (3 : 7) visualized using iodine, IR (KBr) in cm^−1^ *υ* 3250 (N–H); 2895 (Ar–H); 1720 (C=O); 1610 (C=N); 1350 (C–N); 1235 (C–O). ^1^H-NMR (300 MHz, CDCl_3_) *δ*: 2.4 (s, 3H), 3.9 (s, 3H), 4.4 (s, 2H), 6.9 (t, 1H), 7.0 (t, 2H), 7.1 (d, 2H), 7.2–7.7 (m, 8H), 8.0 (s, 1H), 11.7 (s, 1H). MS, m/z: 406 [M^+^_._+1], Anal. Calcd. for C_23_H_23_N_3_O_2_S, Requires: C: 68.12%, H: 5.72%, N: 10.36%, O: 7.89%, S: 7.91%. Found: C: 69.30%, H: 5.00%, N: 10.20%, S: 7.50%.

*Molecule Number 4: 1-(p-Cl-phenyl)-5-(o-methoxyphenyl)-2-S-benzyl Isothiobiuret.* Obtain as off white solid (63.55%) m.p. 122–124°C, TLC *R*_*f*_ 0.8 in EtOAc: Petether (3 : 7) visualized using iodine, IR (KBr) in cm^−1^ *υ* 3250 (N–H); 2850 (Ar–H); 1700 (C=O); 1600 (C=N); 1370 (C–N); 1235 (C–O). ^1^H-NMR (300 MHz, CDCl_3_) *δ*: 3.8 (s, 3H), 4.5 (s, 2H), 7.0–7.2 (m, 5H), 7.3-7.4 (m, 5H), 7.5 (m, 3H), 11.4 (s, 1H). MS, m/z: 427 [M^+^_._+1], Anal. Calcd. for C_22_H_20_ClN_3_O_2_S, Requires: C: 62.04%, H: 4.73%, Cl: 8.32%, N: 9.87%, O: 7.51%, S: 7.53%. Found: C: 58.71%, H: 5.40%, N: 9.00%, S: 7.00%, Cl: 7.50%.

*Molecule Number 5: 1-(o-Cl-phenyl)-5-(o-methoxyphenyl)-2-S-benzyl Isothiobiuret.* Obtain as off white solid (75.14%) m.p. 118–120°C, TLC *R*_*f*_ 0.8 in EtOAc: Petether (3 : 7) visualized using iodine, IR (KBr) in cm^−1^ *υ* 3300 (N–H); 2960 (Ar–H); 1741 (C=O); 1590 (C=N); 1372 (C–N); 1235 (C–O). ^1^H-NMR (300 MHz, CDCl_3_) *δ*: 3.6 (s, 3H), 4.4 (s, 2H), 7.0-7.1 (m, 3H), 7.3-7.4 (m, 8H), 7.5-7.6 (d, 2H), 8.1 (s, 1H), 11.3 (s, 1H). MS, m/z: 427 [M^+^+1], Anal. Calcd. for C_22_H_20_ClN_3_O_2_S, Requires: C: 62.04%, H: 4.73%, Cl: 8.32%, N: 9.87%, O: 7.51%, S: 7.53%. Found: C: 60.40%, H: 4.40%, N: 9.00%, S: 7.00%, Cl: 7.50%.

*Molecule Number 6: 1-(m-Cl-phenyl)-5-(o-methoxyphenyl)-2-S-benzyl Isothiobiuret.* Obtain as off white solid (77.60%) m.p. 105–107°C, TLC *R*_*f*_ 0.8 in EtOAc: Petether (3 : 7) visualized using iodine, IR (KBr) in cm^−1^ *υ* 3280 (N–H); 2900 (Ar–H); 1670 (C=O); 1550 (C=N); 1320 (C–N); 1230 (C–O). ^1^H-NMR (300 MHz, CDCl_3_) *δ*: 3.8 (s, 3H), 4.5 (s, 2H), 6.8–7.1 (m, 4H), 7.3-7.4 (m, 5H), 7.4-7.5 (m, 2H), 7.8-7.9 (m, 2H), 8.3 (s, 1H), 11.5 (s, 1H). MS, m/z: 427 [M^+^+1], Anal. Calcd. for C_22_H_20_ClN_3_O_2_S, Requires: C: 62.04%, H: 4.73%, Cl: 8.32%, N: 9.87%, O: 7.51%, S: 7.53%. Found: C: 65.21%, H: 4.40%, N: 8.70%, S: 7.00%, Cl: 7.60%.

### 2.4. Antimicrobial Activity

All the compounds were screened for their antibacterial activity against pathogenic bacteria and fungi such as* E. coli, S. aureus, P. aeruginosa,* and* Aspergillus fusarium* by cup plate agar diffusion method at a concentration 100 *μ*g/mL in DMSO. The zone of inhibition was measured in mm and is average of three readings. The readings are shown in Table 1.

Molecule 4 showed moderate antimicrobial activity against* E. coli* and* S. aureus,* and considerable antifungal activity, whereas molecule number 2 showed a reverse trend in activities; from this observation, it can be concluded that substitution at para position of phenyl ring plays a crucial role in deciding activity toward bacterial and fungal stains.

### 2.5. Anticancer Activity

Molecule number 1 as representative molecule was studied for short term* in vitro* cytotoxicity using Dalton's ascites (DLA) cells and Ehrlich ascites Carcinoma (EAC) Cells.

The tumor cells aspirated from the peritoneal cavity of tumor bearing mice were washed thrice with phosphate buffered saline (PBS) or normal saline. Cell viability was determined by trypan blue exclusion method, viable cell suspension (1 × 10^6^ cells in 0.1 mL) was added to tubes containing various concentrations of the test compounds, and the volume was made up to 1 mL using PBS. Control tube contained only cell suspension; these assay mixtures were incubated for 3 hours at 37°C. Further cell suspension was mixed with 0.1 mL of 1% trypan blue and kept for 2-3 minutes and loaded on a haemocytometer. Dead cells take up the blue colour of trypan while live cells do not take up the dye. The numbers of stained and unstained cells were counted separately; drug concentration versus percentage of death cells was tabulated in Table 2:

(1) %Cytotoxicity =Number  of  dead  cellsNumber  of  live  cells+Number  of  dead  cells×100.

## 3. Conclusions

From the observation, it can be concluded that substitution at para position of phenyl ring plays a crucial role in deciding activity toward bacterial and fungal stain; as these molecules are easy to synthesize and purify, these classes of molecules can be explored further to develop SAR against different microbial and fungal stains as well as a potent anticancer agent.

## Figures and Tables

**Table 1:** Antimicrobial activities of molecules numbers 1 to 6.

Molecule number	Antibacterial activity			Antifungal activity
	*E. coli *	*S. aureus *	*P. aeruginosa *	*Aspergillus fusarium *
1	12	8	15	10
2	18	18	19	12
3	18	10	10	10
4	12	12	8	18
5	10	17	18	10
6	10	18	16	8
Amikacin	25	23	23	NA
Ketoconazole	NA	NA	NA	23
Control (DMSO)	Nil	Nil	Nil	Nil

Including well diameter of 5 mm.

**Table 2:** Anticancer activity of molecule number 1.

Compound number	Drug concentration (*μ*g/mL)	Percentage cell death (DLA)	Percentage cell death (EAC)
Molecule 1	200	64	70
	100	40	56
	50	26	36
	20	13	16
	10	6	6

5-Fluorouracil	100	NA	92
	50	97	NA
	20	NA	29
	10	24	NA

5-Fluorouracil was used as a standard. Molecule 1 shows considerable cell toxicity at 50 and 20 *μ*g concentration.
